# Lactic Acid Bacteria from African Fermented Cereal-Based Products: Potential Biological Control Agents for Mycotoxins in Kenya

**DOI:** 10.1155/2022/2397767

**Published:** 2022-02-22

**Authors:** Eliud N. Wafula, Christabel N. Muhonja, Josiah O. Kuja, Eddy E. Owaga, Huxley M. Makonde, Julius M. Mathara, Virginia W. Kimani

**Affiliations:** ^1^Department of Public Health, Bomet University College, P.O. Box 701-20400, Bomet, Kenya; ^2^Department of Biological Sciences, Machakos University, P. O. Box 136–90100, Machakos, Kenya; ^3^College of Graduate Studies and Research, Mount Kenya University, P.O Box 342-01000, Thika, Kenya; ^4^Institute of Food Bioresources Technology, Dedan Kimathi University of Technology, P.O. Box 657-10100, Nyeri, Kenya; ^5^Department of Pure & Applied Sciences, Technical University of Mombasa, P.O Box 90420-80100, Mombasa, Kenya; ^6^Department of Food Science and Technology, Jomo Kenyatta University of Agriculture and Technology, P.O. Box 62000-00200, Nairobi, Kenya; ^7^Division of Industrial Microbiology and Biotechnology, Kenya Industrial Research and Development Institute, P.O Box 30650-00200, Nairobi, Kenya

## Abstract

Cereals play an important role in global food security. Data from the UN Food and Agriculture Organization projects increased consumption of cereals from 2.6 billion tonnes in 2017 to approximately 2.9 billion tonnes by 2027. However, cereals are prone to contamination by toxigenic fungi, which lead to mycotoxicosis. The current methods for mycotoxin control involve the use of chemical preservatives. However, there are concerns about the use of chemicals in food preservation due to their effects on the health, nutritional quality, and organoleptic properties of food. Therefore, alternative methods are needed that are affordable and simple to use. The fermentation technique is based on the use of microorganisms mainly to impart desirable sensory properties and shelf-life extension. The lactic acid bacteria (LAB) are generally regarded as safe (GRAS) due to their long history of application in food fermentation systems and ability to produce antimicrobial compounds (hydroxyl fatty acids, organic acids, phenyllactic acid, hydrogen peroxide, bacteriocins, and carbon dioxide) with a broad range of antifungal activity. Hence, LAB can inhibit the growth of mycotoxin-producing fungi, thereby preventing the production of mycotoxins. Fermentation is also an efficient technique for improving nutrient bioavailability and other functional properties of cereal-based products. This review seeks to provide evidence of the potential of LAB from African fermented cereal-based products as potential biological agents against mycotoxin-producing fungi.

## 1. Introduction

Africa is an origin and the major producer of numerous cereals, including maize, finger millet, sorghum, pearl millet, rice, and teff [1]. Cereal consumption accounts for more than 50% of worldwide caloric daily intake [2], while in Eastern Africa, cereals consumption accounts for over 40% of the total human dietary calorie intake [3]. Maize is the major cereal crop produced and consumed in sub-Saharan Africa (SSA), with about 300 million people depending on it for livelihood [1]. It is the most important staple food crop relied upon by approximately 96% of Kenyan citizens [4]. On average, maize consumption in Kenya stands at 400 g per person per day, with 98 kg annual consumption per capita [5].

The chemical nature of cereal grains predisposes them to fungal contamination and infection, especially during production, processing, and storage [6]. Although earlier data from Food and Agricultural Organization (FAO) estimate mycotoxins contamination of food crops globally to be 25%, more recent studies from the European Food Safety Authority (EFSA) show the prevalence of detectable mycotoxins could be up to 60–80% [7]. Among the cereals, maize is the most prone to attack by potentially harmful opportunistic fungi, which may lead to mycotoxin contamination [8].

The growth of fungi lowers grain quality and yield leading nutritional and chemical changes. Specifically, it results in grain discolouration, a loss in dry matter due to carbohydrate utilization, protein, and lipid degradation, which result in changes in product digestibility and volatile metabolites production that gives off odour [9]. Furthermore, the production of heat and moisture in cereals during mould infestation contributes to product spoilage, which affects grain grade, marketing price, and customer satisfaction. The major concern is the production of toxic secondary fungal metabolites (mycotoxins) with adverse health effects on humans and animals [10]. Mycotoxins are mostly produced by fungi of genera *Aspergillus*, *Penicillium, Alternaria*, and *Fusarium*, affecting cereal grains during growth or after harvesting [11]. Genus *Fusarium* has been implicated as a cause of mycotoxin contamination in grapes through production of a variety of mycotoxin metabolites [12].

The extent of mycotoxin contamination in tropical regions can be attributed to the warm and moist weather, poor agronomic practices, inadequate conditions of crop storage, and weak regulatory systems during food handling and processing [13–15]. Aflatoxin is the most commonly reported mycotoxin in tropical developing countries [5] and is mainly produced by *Aspergillus flavus* and *A. parasiticus* [10], with significant public health importance due to its effects on human health [16].

The commonly used methods in mycotoxin control include chemical preservatives for the prevention of mould growth [17], decontamination of foods and feeds, and surveillance of mycotoxins in crops, foods, and feeds [18]. However, the use of chemical preservatives has raised food safety concerns. Furthermore, the lack of strong regulatory systems in most African countries has contributed to recurrent mycotoxicosis [15, 19]. This has triggered the investigation of novel antifungal agents produced by various lactic acid bacteria (LAB) strains as potential natural alternatives [20]. Among the strategies currently being considered for food, preservation is the use of microorganisms commonly occurring in fermented foods or their antimicrobial metabolites [21]. The use of natural food preservation methods is regarded as healthy, safe, and friendly by consumers, besides having a lower impact on food nutrition and organoleptic properties [22]. These technologies are cost-effective since they do not require advanced skills or equipment hence can be adopted in developing countries [21].

The use of LAB has shown the potential to control the growth of mycotoxins-producing fungi, thereby preventing mycotoxin production [23]. LAB are generally regarded as safe due to their long history in food fermentation [24]. They are known to produce nontoxic metabolites with broad-spectrum activity [25]. Fermented food can act as vehicles or reservoirs of beneficial LAB that produce antifungal products, which can help in food preservation and control of toxigenic fungi. Therefore, this review seeks to provide evidence of the potential of LAB from African fermented cereal-based products as potential biological agents against mycotoxins-producing fungi. This is important as it builds on indigenous knowledge in food production and preservation systems, which is in line with the African commission priority areas of Agenda 2063 framework and the United Nations sustainable development goals relating to improved food and nutrition security [26, 27].

## 2. Types and Distribution of Mycotoxins in Africa

Mycotoxins are secondary metabolites produced by fungi and are poisonous to both humans and animals. Worldwide, five mycotoxins are considered to be economically and toxicologically important: fumonisins (FUMs); aflatoxins (AF); ochratoxins A (OTA); deoxynivalenol (DON) and derivatives (DON-3-glucoside, monoacetyl-DONs, norDONs, and deepoxy-DON); zearalenone (ZEA) and derivatives [28]. Other mycotoxins of interest include patulin, T-2 toxin, moniliformin (MON), nivalenol (NIV), enniatins, beauvericin, and HT-2 toxin [28]. In Africa, the most prevalent mycotoxins are the AFs (43.75%), followed by FUMs (21.87%), OTA (12.5%), ZEA (9.375%), NIV, and beauvericin (both at 6.25%) [29], as indicated in Table 1. In SSA and specifically East Africa, aflatoxins are the most commonly reported mycotoxins, with previous episodes of AF poisoning having been documented in Kenya, Ethiopia, Uganda, and Tanzania [29–32].

Aflatoxins are a group of 20 related fungal metabolites that are classified into four main categories, namely, aflatoxins *B*_1_, *B*_2_, *G*_1_, and *G*_2_ [33]. Aflatoxins *M*_1_ and *M*_2_ are metabolites of aflatoxins *B*_1_ and *B*_2_, respectively, occurring in milk and milk products from animals consuming feeds or humans consuming aflatoxins *B*_1_ and *B*_2_ contaminated foods [34]. Maize, groundnuts, and tree nuts are the most common foods at risk of aflatoxins contamination [35], as shown in Table 1. Among the fumonisins, fumonisins *B*_1_, *B*_2_, and *B*_3_ are the major forms found in food [33]. The most common animal feeds harbouring mycotoxins include maize grain, wheat bran, pea hulls, and noug cake [18]. As indicated in Table 1, *Aspergillus flavus*, *A. parasiticus,* and *A. nomius* are some of the most common aflatoxigenic fungi [35]. Besides, other reported aflatoxigenic fungi include *A. bombycis*, *A. ochraceus,* and *A. pseudotamari* [35]. *Aspergillus* spp. are widely found in soil used for growing crops, processing facilities, storage areas, and the distribution systems for manufactured products. *A. flavus* strains have variable aflatoxin capabilities ranging from nontoxigenic to highly toxigenic but often produce more aflatoxin *B*_1_ than aflatoxin *G*_1_ [35]. On the other hand, there is less variation in toxigenicity among *A. parasiticus* strains and produce aflatoxin *B*_1_ and different amounts of aflatoxins *B*_2_, *G*_1_, and *G*_2_ [18]. *Fusarium* fungi are major sources of trichothecene mycotoxins, while other genera that produce trichothecene include *Myrothecium, Stachybotrys, Cylindrocarpon, Trichothecium, Cephalosporium, Trichoderma, Verticimonosporium,* and *Phomopsis* [36].

Zearalenone is a potent estrogenic metabolite produced by some *Gibberella* and *Fusarium* species such as *F. graminearum*, *F. culmorum*, *F. verticillioides*, *F. incarnatum, F. semitectum*, *F. equiseti, F. oxysporum,* and *F. sporotrichioides* [18]. Zearalenone often cooccurs with deoxynivalenol in cereal grains [7, 37]. Fumonisins B_1_ and B_2_ are naturally occurring mycotoxins produced by *F. verticillioides*. Aflatoxins and fumonisins are common contaminants of maize, but to a lesser extent of rice, sorghum, wheat, and cereal-based foods prepared from these commodities [33]. Cases of coexposure can either occur from the same food being contaminated with both mycotoxins or within the diet from different foods, each contaminated with one or the other [33].

Ochratoxins and citrinin are produced mainly by members of the genera *Aspergillus* and *Penicillium*. Ochratoxin A (OTA) is produced by two species: *A. ochraceus* and *P. verrucosum*. These fungi are ubiquitous and have great potential for contamination of animal feed and human food [38]. OTA often occurs in most cereal grains (wheat, oats, corn, and barley), cheese, peanuts, grapes/raisins, and dried fruits. Since it has a long half-life, OTA accumulates in the food chain. Citrinin cooccurs with OTA and often contaminates cereal grains (such as barley, corn, oats, wheat, and rice), peanuts, and fruits [38]. The fungal genera *Aspergillus, Penicillium, Neotyphodium,* and *Claviceps* can produce tremorgenic mycotoxins [39]. Fungi that produce tremorgens grow on dairy, stored grains and nuts (walnuts and peanuts), and forages [38, 39].

### 2.1. Aflatoxicosis Prevalence in Kenya

Episodes of aflatoxin contamination and poisoning have been reported in different parts of Kenya. The worst outbreak of aflatoxins poisoning was in 2004 when 317 cases were reported and 125 people died in the Makueni and Kitui districts in Eastern Kenya [40, 41]. There was another aflatoxicosis outbreak in 2005 in the same district of Makueni and Kitui affecting 75 people, resulting in 32 deaths [42]. Additionally, an aflatoxin outbreak occurred in 2010 during the season of abundant rainfall, which resulted in a good grain yield in Eastern Kenya [43]. The Kenyan Government banned the consumption and trade of approximately 200,000 tons of maize to protect the public from mycotoxicosis [43].

A study to compare aflatoxin prevalence in lowland and highland areas of Makueni county, Kenya, showed that low-altitude areas had more aflatoxin-contaminated maize than high-altitude areas [44]. About 50% of positive aflatoxin-contaminated maize samples in lowlands had aflatoxin levels exceeding 10 ppb, while in highlands, none of the maize had aflatoxin contamination exceeding 10 ppb [44]. An analysis of aflatoxin contamination in maize, millet, and sorghum across five counties of Kwale, Isiolo, Tharaka-Nithi, Kisii, and Bungoma indicated that about 20% of all maize samples had aflatoxin contamination levels above Kenyan legal limits of 10 ppb [45]. Consequently, the study revealed the mean aflatoxins level of 1.12 ppb in millet, hence the need to consider the potential role of traditional cereals such sorghum and millet in contributing to aflatoxin exposure in East Africa.

In a study to determine levels of aflatoxin-producing fungi and aflatoxin contamination of relief maize meal and a nutritional supplement of maize (Unimix) in Northern Kenya, the prevalence of *Penicillium* and *Fusarium* spp. occurred at exceptionally higher levels than the targeted aflatoxin-producing fungal spp.: *A. parasiticus* [46]. This finding implied that dietary coingestion of ochratoxins, fumonisins, trichothecenes, and zearalenone alongside aflatoxins is an unfortunate possibility among the residents of Northern Kenya. In children below the age of 5 years in Nandi and Makueni counties, the aflatoxin exposure rate from maize consumption was 0.011 and 0.49 ppb body weight per day, respectively. Consequently, aflatoxin exposure through milk was 4 × 10^−4^ and 1 × 10^−4^ ppb body weight per day in Nandi and Makueni counties, respectively [47]. Stunting growth and severe stunting growth rates associated with aflatoxin exposure in Makueni and Nandi were 28.7% and 18.5%; 30.7% and 16.5%, respectively. In 2004, the number of children who were wasting and fed on aflatoxin-contaminated flour in the Kisumu district was highly significant [47]. According to Tola and Kebede [18], the concentrated animal feedstuffs contain the highest level of mycotoxins. For example, 7 ppb was the lowest level of aflatoxin B_1_ contamination recorded from silage feed, but in concentrated animal feeds like wheat bran, noug cake, and sweet pea hull, the highest level of aflatoxin B_1_ contamination was about 419 ppb [18].

### 2.2. Aflatoxin Levels in Foods and Feeds in Kenya

Cereals are widely cultivated and consumed in Africa; however, they are most sensitive to aﬂatoxin contamination. A study by Sirma et al. [32] indicated that 60% of all maize samples (269) analysed from Laboret, Kilibwoni, and Chepkongony in Kenya were contaminated with aflatoxins ranging between 0.17 and 5.3 ppb. Similarly, 92.3% of all millet samples [39] collected from the study sites recorded aﬂatoxin levels ranging from 0.14 to 6.4 ppb. On the contrary, 37% of all sorghum samples [48] analysed recorded aﬂatoxin levels beyond the Kenya Bureau of Standards (KEBS) maximum tolerable limit of 10 ppb. Kilibwoni sublocation had the highest percentage (46%), while both Laboret and Chepkongony had 27.3%, each of samples exceeding the maximum tolerable limit of 10 ppb [32].

A study conducted in 2004 during the major aflatoxin outbreak in Kenya ascertained that 55% of maize products had aflatoxin levels greater than the Kenyan regulatory limit of 10 ppb, 35% had levels >100 ppb, and 7% had levels >1,000 ppb [49]. Locally obtained maize from the affected area was significantly more likely to have aflatoxin levels >10 ppb than maize bought from other regions of Kenya or other countries [49]. In a survey of aflatoxin contamination of milk and animal feeds (mostly cereal-based) across urban centres in Kenya, 86% of feed samples from farmers were positive for aflatoxin B_1_, while 81% of feed samples from feed millers and 87% from agrochemical shops were positive for AFB_1_ with 67%, 58%, and 66%, respectively [50]. Further, the positive samples exceeded the FAO/WHO level of 5 ppb. Standards for allowable aflatoxin limits in food for human consumption vary from country to country. For instance, the allowable level is 4 ppb in France and the Netherlands, 5–20 ppb in Canada and the USA, and 30 ppb in India, as reported by James et al. [51]. A range of 4–30 ppb is widely recognized as the acceptable limit of aflatoxin in food [52]; 20 ppb is the internationally recommended maximum limit of aflatoxin contamination. In Kenya, the maximum allowable limit in foods and feeds by the Kenya Bureau of Standards (KEBS) is 10 ppb and 20 ppb, respectively [53].

### 2.3. Mycotoxins Effects on Human and Animal Health

Mycotoxicoses in humans can be categorized as acute (rapid onset and a clear toxic response) or chronic (low dose exposure over a long period), leading to cancer and other health effects [28]. AFB_1_ is classified as a class 1 human carcinogen [54]. Chronic exposure to AFB_1_ has been linked to the development of liver cancer in humans [55]. Aflatoxin is the third most leading cause of hepatocellular carcinoma or liver cancer deaths globally, with about 83% of these reported deaths occurring in sub-Saharan Africa and East Asia [15, 56].

In Kenya, estimates for hepatocellular carcinoma (HCC) were associated with aflatoxin ranges from 1.05 to 39.9 persons per 100,000, but globally aflatoxin may play a causative role in 4.6–28.2% of all global HCC [57]. It is reported that aflatoxin can be metabolized by specific P450 enzymes in the liver into aflatoxin-8,9-epoxide (a reactive oxygen species) that then either binds to proteins and causes acute toxicity (aflatoxicosis) or binds to DNA leading to lesions that over time increase the risk of HCC [58]. Aflatoxin exposure (low to moderate levels) has been associated with stunting in children [16, 59], congenital malformations, and immunosuppression associated with teratogenic effects [52]. There is also strong evidence linking aflatoxin with reproductive disorders such as infertility, reduced sperm quality, and increased rate of stillbirths in both humans and animals [15, 60].

A study by Wakhisi et al. [61] found high incidences of oesophagal cancer in the North Rift valley that was associated with fumonisin exposure. Fumonisins, however, are thought to be possible cancer promoters of aflatoxin carcinogenicity [33]. Reports show some evidence and concern that additive or synergistic actions occur when there is coexposure of fumonisins and aflatoxins that potentially increase carcinogenicity [62]. The exposure of nursing children to aflatoxin through breast milk was 6 × 10^−3^ and 1 × 10^−6^ ppb per kg body weight per day in Makueni and Nandi, respectively, and children below the age of 30 months in Makueni had 1.4 times higher levels of aflatoxin M_1_ (AFM_1_) in urine than those of the same age in Nandi [47].

Health effects occur in animals, livestock, and poultry since aflatoxins are potent hepatotoxins, immunosuppressants, mutagens, and carcinogens [18]. It is reported that milk, beef, or wool reproduction and growth can be altered when ruminants consume mycotoxin-contaminated feed for extended periods [48]. In poultry, aflatoxins have been reported to cause liver damage, impaired productivity and reproductive efficiency, decreased egg production, inferior eggshell quality, inferior carcass quality, increased susceptibility to diseases, stunted growth, and death [33]. In pigs, aflatoxins are mainly associated with liver damage, whereas in cattle, it leads to reduced weight gain, liver and kidney damage, and reduced milk production [33]. Published information indicates that different forms of enzymes (such as cytochrome P450s and glutathione S-transferases), which metabolize aflatoxins, are considered responsible for the susceptibility of different animals to the toxic effects of aflatoxins [33]. Citrinin acts as a nephrotoxin in some animal species but with variation in acute toxicity in different species. The 50% lethal dose for ducks, chickens, and rabbits is 57 ppb, 95 ppb, and 134 ppb, respectively [35]. Citrinin can act synergistically with OTA to depress RNA synthesis in murine kidneys [35, 63]. Tremorgenic mycotoxins are known to elicit either intermittent or sustained tremors invertebrate species [39]. Zearalenone is linked to reproductive problems in cattle and sheep [64]. Dietary concentrations of zearalenone at 1,000 ppb can lead to hyperestrogenic syndromes in pigs, but higher concentrations can result in disrupted conception and abortion [35].

## 3. Control of Moulds' Contamination and Mycotoxins

### 3.1. Regular Surveillance of Mycotoxins in Food and Feedstuﬀs and Awareness Creation

Food grains and animal feeds need to be harvested correctly, dried completely, and stored properly. African countries need to strengthen nationwide surveillance and increase food and feed inspections to ensure food safety and local education and assistance [18]. Most smallholder farmers lack knowledge on mycotoxin contamination of staples and their health impacts in Africa [65, 66]. Surveys conducted in South Africa showed that people used fungi-contaminated staples for food, implying that they are not fully aware of the health hazards associated with the ingestion of mycotoxins [65]. Alberts et al. [67] noted that the lack of effective and sustained awareness and education of the threat of mycotoxins to human health hinders any mitigation strategy. To find appropriate means to prevent fungal infections of crops, education and training to raise awareness of smallholder farmers and consumers on mycotoxins are important [65, 66]. This could be achieved by creating awareness of the harmful effects of mycotoxins on human and animal health and productivity. The involvement of the government, private organizations, nongovernmental organizations, and national media networks and organizing seminars, workshops, and features in newspapers and magazines are needed to ensure local sustainability of mycotoxin interventions [68]. However, such important continuous efforts are impeded by inadequate research funding and technology in many research institutions that facilitate this surveillance in various African countries and the lack of adequate local experts on this problem [29]. Therefore, both local and international efforts must complement each other to overcome the mycotoxin menace in Africa.

### 3.2. Preharvest and Postharvest Prevention of Fungal Growth in Crops and Other Feedstuﬀs

Good Agricultural Practices (GAP) also play a critical role in reducing aflatoxin levels in foods. Proper land preparation, early planting, crop rotation, weeding, correct spacing, cleaning stores, sorting maize after shelling, use of wooden pallets, and stooking were indicated as GAP that led to reduced levels of aflatoxin [69]. Proper drying and storage of crops can eﬀectively reduce mould growth and mycotoxin production. Diversification of crop varieties is also a recommended practice in reducing aflatoxin levels in harvested foods. A higher occurrence of aflatoxin was associated with monocropping systems, subhumid agroecological zone, and samples with broken kernels [43]. Prevention of mould growth can be achieved by following strict hygienic protocols during harvesting, storage, and processing of crops and feeds. It has been demonstrated that early harvesting of groundnuts resulted in lower aﬂatoxin levels [29]. Analysis of paired grain samples (visually sorted and unsorted) showed that sorting reduced fumonisin by 65%. For instance, an intervention study at subsistence farms conducted in the lower Kindia region of Guinea reported a 60% reduction in mean aflatoxin levels was achieved by thorough drying and proper storage of groundnuts [70]. These studies indicate that the timing of rainfall, rather than the total amount of rainfall, might be important in determining the spatial risk of aflatoxin accumulation.

In some African countries, a high percentage of calories come from maize, which is commonly contaminated by aflatoxins and fumonisins. Besides, the consumption of peanuts is another major source of exposure to aflatoxin [68]. The lack of dietary diversity is directly related to high levels of mycotoxin exposure [71]. Therefore, access to a wider variety of foods and the replacement of those at high risk of contamination will lower the risk of exposure [58].

### 3.3. Decontamination of Mycotoxin

#### 3.3.1. Physical Approaches

Sorting, washing, and crushing combined with dehulling of maize grains, were eﬀective in the removal of aﬂatoxin and fumonisin in Benin [72]. Traditional food processing methods are sustainable, practical, and inexpensive postharvest intervention strategies to reduce mycotoxin contamination and exposure [67]. Hand sorting and segregation of crops before cooking is a common practice in many countries in Africa [66, 67]. The postharvest practices for maize include shelling, winnowing, dehulling, and milling [67]. A significant reduction of fumonisins has been demonstrated in several African countries through effective hand sorting of maize [66]. A study of the acoustic-based screening method for mycotoxin contamination indicated a linear inverse relationship between aflatoxin contamination and the amplitude of the acoustic signal penetrating the nut and corn samples [73].

#### 3.3.2. Chemical Approaches

Fungicides, such as cyproconazole, epoxiconazole, tebuconazole, oltipraz, prochloraz, propiconazole, azoxystrobin, and chlorophyllin, have been used to control *Fusarium* head blight for reduction of fumonisin and aﬂatoxin contamination [29]. Chemoprotection against aflatoxin has been employed with the use of several chemical compounds, such as oltipraz and chlorophyllin [29]. Dietary substances, including green tea and broccoli sprouts, have exhibited increased detoxification processes in animals and are being considered as effective approaches for reducing the health hazards caused by various mycotoxins [74]. For example, the aflatoxin-induced changes in the liver of mice were significantly reduced with the cotreatment of black tea extract [75]. The introduction of technologies for specific, efficient, and environmentally sound prevention of mycotoxins is inevitable [76]. For instance, the use of improved crop varieties such as maize genotypes with some resistance to fumonisin accumulation has been identified [77]. It is reported that transgenic Bt maize is less prone to insect damage and fumonisin accumulation than non-Bt hybrids [78], but the effectiveness of Bt maize in reducing aflatoxin contamination is still unclear [67].

#### 3.3.3. Biological Methods

Most of the biological control strategies are promising and have shown reduced concentrations of aflatoxins, but the structure and toxicity of the detoxified products remain unclear [76]. Such strategies include breeding of maize-resistant cultivars, the introduction of biocontrol microorganisms, application of phenolic plant extracts, and expression of antifungal proteins and mycotoxins degrading enzymes in transgenic maize cultivars for the development of atoxigenic fungi that compete with toxigenic fungi in the environment [67]. The studies on microbial binding mainly focus on probiotics strains of lactic acid bacteria (LAB), including species of *Lactobacillus, Lactococcus*, *Bifidobacterium* spp., and *Propionibacterium* and yeast strains of *Saccharomyces cerevisiae* [76].

## 4. Degradation of Mycotoxins

Different approaches have been developed for the elimination and reduction of mycotoxins in foods and feeds, including physical, chemical, and biological methods. These methods work through modification and destruction of the toxins molecular structure, thereby inhibiting their transfer and absorption in the digestive system and thus reducing toxin's accessibility to the target tissue and eventually removing it [79, 80]. The physical methods include extrusion cooking, which is broadly used in the food industry, especially for cereals and cereal foodstuffs. It is a process that combines exposure to high temperature and high pressure in a very short time [81]. This method can destroy or inactivate aflatoxin and deoxynivalenol in maize flour [82]. The use of hydrated sodium calcium aluminosilicates is another novel and effective strategy to reduce the bioavailability of aflatoxins [83]. Microwave heating, ozone, gamma, and electron beam irradiation, ultraviolet and pulsed light, electrolyzed water, and cold plasma treatments are other good examples of physical methods for mycotoxins detoxification [84]. The drawbacks of physical methods include negative effects on food quality, safety, and human and animal health. The use of high temperature and pressure destroys nutrients in foods such as water-soluble vitamins and proteins.

The chemical strategies used in mycotoxins detoxification include ammoniation, which is regarded as the most advanced and economically practicable technique to detoxify aflatoxins in foodstuffs. Ammoniation treatment has been developed to detoxify ochratoxin A (OTA) in animal feed and alcoholic beverages [85]. Other chemicals that have shown maximum degradation rate in cereals include sodium sulfite, sodium hydrogen sulfate, sodium hydroxide, sodium hypochlorite, and hydrogen peroxide [86]. Neutral electrolyzed oxidizing water (NEW) is one of the two products produced during the process of electrolysis of pure water; this method could potently reduce the content of AFB_1_ in peanuts [87]. Unfortunately, chemical residues are generally unsafe and economically unrealistic for commercial applications [82].

The biological detoxification of mycotoxins involves fermenting microorganisms and/or enzymes to degrade or transform mycotoxins into less toxic compounds [88]. Various microorganisms have been reported to have the capacity for mycotoxins transformation and detoxification, for example, bacteria (e.g., LAB), yeast, and some moulds [89]. These microorganisms act in the intestinal tract of animals before resorption of the mycotoxins. Biological transformation reactions include acetylation, glycosylation, ring cleavage, hydrolysis, deamination, and decarboxylation [80]. The use of LAB in food fermentation is generally regarded as safe (GRAS) for consumption, they dominate many food fermentation systems and they produce metabolites with antifungal properties [17]. Therefore, this is the most encouraged method for mycotoxins biodetoxification of contaminated agricultural products [90]. Two mechanisms for AF detoxification by microbial methods, i.e., cell wall component adhesion and microbial enzymes, were studied and different LAB species were found to have varied effectiveness of AF detoxification in different food samples [91]. A combination of extrusion and fermentation with two LAB strains was confirmed as a promising innovative pretreatment for wheat bran by enhancing its nutrition value and safety [92].

### 4.1. LAB from African Fermented Cereal Foods

**Figure 1:**
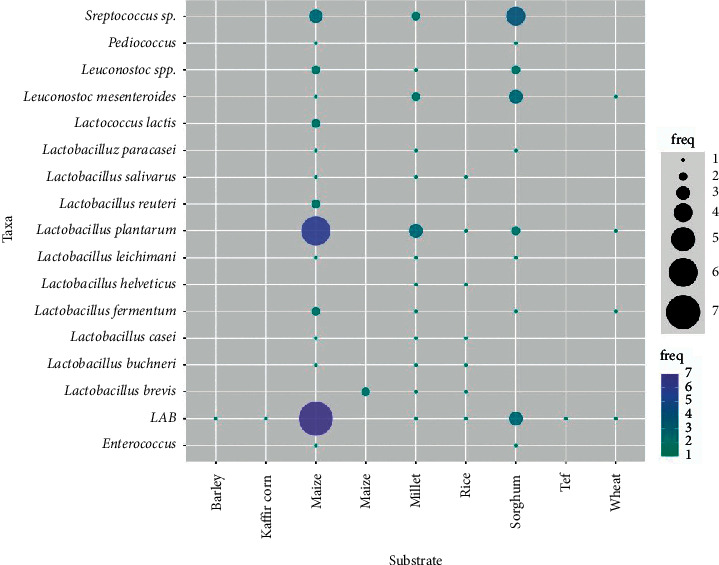
The most commonly used LAB for cereal-based fermentation in Africa (freq = frequency of usage). Adopted with modifications from [98–105].

Traditionally, LAB are used as starter cultures for dairy, vegetable, cereal, and meat fermentations because of their contribution to flavour development and preservative potential [93, 94]. The commonly used LAB belong to the genera *Lactobacillus*, *Lactococcus*, *Leuconostoc*, *Oenococcus*, *Pediococcus, Streptococcus*, *Tetragenococcus*, *Aerococcus*, *Carnobacterium*, *Enterococcus*, *Vagococcus*, and *Weissella* [95]. The inhibitory activity of LAB over moulds may result from the production of metabolites such as organic acids (lactic, propionic, and acetic acids), carbon dioxide, ethanol, hydrogen peroxide, diacetyl, and other low-molecular-weight peptides [96, 97]. Other potential underlying mechanisms include competitive growth exclusion, a decrease in the pH caused by acid production, or a combination of these factors [96]. Various LAB are used in the fermentation of cereal-based African foods (Table 2 and Figure 1). The success in producing fermented functional food products is due to the industrial robustness of LAB. Industrially, the probiotic nature of LAB is supplemented with standard gene functions, which improve the activity of LAB under hostile processing conditions [106].

A study by Moroni et al. [107] reported a broad spectrum of LAB belonging to the genera *Lactobacillus, Pediococcus,* and *Leuconostoc* in stable sourdoughs. Buckwheat and teff sourdoughs were dominated mainly by obligate or facultative heterofermentative LAB, which are commonly associated with traditional wheat or rye sourdoughs [108]. Similarly, the spontaneous fermentation of the gluten-free flours resulted in the selection of species such as *Pediococcus pentosaceus, Leuconostoc holzapfelii, Lactobacillus gallinarum, L. vaginalis, L. sakei, L. graminis*, and *Weissella cibaria* that are not endemic to traditional sourdoughs [107]. Moroni et al. [107] also reported *L. plantarum* and *L. pontis* as the dominant species in all buckwheat and teff sourdough, respectively. A study by Nwachukwu et al. [109] showed various LAB (*L. plantarum*, *L. pentosus*, *L. cellbiosus*, *Pediococcus pentosaceus,* and *Leuconostoc mesenteroides*) are involved in the spontaneous fermentation of maize, sorghum, and millet for the production of Nigerian indigenous foods (akamu). Moreover, *W. cibaria* was detected in both spontaneous and starter-induced type fermentations of buckwheat [107]. *Pediococcus* spp. are, however, regarded as endemic in Ethiopian traditional fermented foods (such as injera) produced from teff flour. Elsewhere, Corsetti et al. [110] isolated *L. graminis* from wheat grains.

Members of four LAB genera (*Lactobacillus, Leuconostoc, Enterococcus*, and *Streptococcus*) were reported in “*kpètè-kpètè*,” a traditional beer produced in Benin [111]. Moreover, five species of Lactobacillus (*L. fermentum, L. divergens, L. bifermentum, L. fructivorans*, *L. casei,* and *L. acidophilus*) were identified in the same study. In another study, species of the genus *Lactobacillus* (*L. divergens, L. fermentum, L. bifermentum, L. fructivorans, L. viridescens, L. hilgardii, L. kandleri,* and *L. casei*) were reported as dominant in traditional beer “*tchoukoutou*” [112]. According to Vieira-Dalodé et al. [113], *L. fermentum* was the most predominant species; however, also the presence of *Leuconostoc mesenteroides* was noted during the process of “*gowé*” fermentation. Likewise, *L. fermentum* was found to dominate in “bushera,” a Ugandan traditional fermented beverage [104].

In Africa, the fermentation technology is based on the ability of LAB to break down complex molecules (substrate) into simpler compounds as the preferred functional products (Table 2 and Figure 1). Maize, millet, and sorghum are the common substrates for the fermented product, with *L. plantarum* as the most preferred LAB, followed by *Leuconostoc mesenteroides* (Figure 1). Other LAB are also useful and mostly used in combination with other stable species to produce a blend of a functional product. Microbial fermentation coupled with biotechnology and biotechnological processing techniques provides a variety of tools to modify cereal products.

#### 4.1.1. Mechanism of Mycotoxin Detoxification by LAB

**Figure 2:**
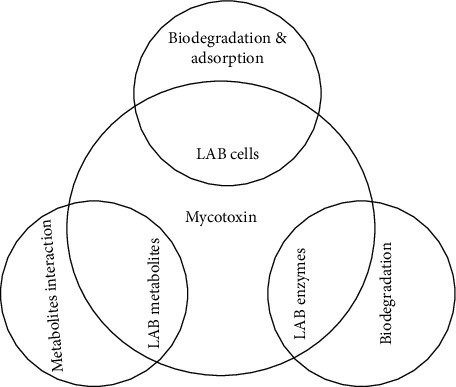
Mechanisms of mycotoxin detoxification by lactic acid bacteria.

Mycotoxins detoxification by LAB is achieved through either the viable cell, their enzymes, or the bioactive metabolites they produce, resulting in the limitation of the growth of fungi and prevention of the production of mycotoxins in food [114], as illustrated in Figure 2. The metabolites produced work via binding and absorption of mycotoxins onto the bacterial cell walls and utilizing them as primary sources of carbon, nitrogen, and phosphorus in the production of enzymes responsible for the degradation (Table 3) [136]. Aflatoxins are also removed by LAB through the biodegradation/bioadsorption mechanisms via direct binding based on probiotic affinity toward aflatoxins [137–139]. Biodegradation of modified AFB_1_ structure can result in undesirable metabolites (such as aflatoxin), which are probably harmful to the host [137–139]. The activities of aflatoxin toxicity are affected by two sites of furofuran and lactone rings. Any variations in the coumarin structure can change mutagenic properties of the aflatoxins; during aflatoxin detoxification, the difuran ring of the mycotoxin is cleaved usually by specific microorganisms such as *Lactobacillus* spp. and *Bifidobacteria* spp. or their enzymatic metabolites [138], as indicated in Figure 2. Previous reports indicate that AFB_1_ can be removed by probiotic bacteria through physical adhesion and binding to the carbohydrate components of the bacterial cells [140].

Many LAB strains are safe microorganisms, easy to grow on a large scale, and a common byproduct of the food industry. They also have a great capacity for adsorption and/or uptake of various kinds of chemical contaminants that are present in an aqueous solution. The mechanisms of interaction of LAB with food contaminants are varied (Figure 2) and depend on the nature of the contaminant, the microbial strain, and the physicochemical conditions [141].

### 4.2. Natural Antifungal Compounds Produced by LAB

The growth of fungi and the production of mycotoxin on food and feed is the major cause of food poisoning and human health concerns. The use of fungicides and other chemicals in food preservation negatively affects health and the environment. However, biopreservative agents, especially from LAB, have been reported to be safe, effective, and biodegradable and can confer health benefits to consumers [142]. Furthermore, Shehata et al. [142] showed broad antimicrobial activity of cell-free supernatant of certain members of *Lactobacillus* spp. against toxigenic fungi. As shown in Table 4, various active antimicrobial compounds are produced by LAB during bacterial food fermentation, for example, hydroxyl fatty acids, lactic, benzoic, and acetic acids, ethanol, carbon dioxide, hydrogen peroxide, and bacteriocins [142]. Various members of *Lactobacillus* spp. have been reported to produce novel antimicrobial compound 3-phenyllactic acid (PLA) with broad-spectrum antifungal activity against different food spoilage and toxigenic moulds, such as *Aspergillus flavus* and *Penicillium expansum* [143]. LAB properties to inhibit pathogens have been associated with the low acidity of the fermentable substrates [166].

Studies by Ahlberg et al. [167] showed that Kenyan traditionally fermented maize and milk-based products contain various *Lactobacillus* strains with growth inhibitory activities against aflatoxin-producing *A. flavus* moulds. Literature indicated that L. *plantarum* 21B isolated from sourdough showed antifungal activity against different toxigenic and food spoilage moulds [145]. The study further showed that the antifungal compounds responsible for such antifungal activities included phenyllactic and 4-hydroxy-phenyllactic acids [145]. Additionally, various researchers have shown that *L. coryniformis, L. casei, L. amylovorus*, and *L. plantarum* produce antifungal cyclic dipeptides, such as cyclo(Phe-Pro), cyclo(Phe-OH-Pro), cyclo-(Leu-Pro), 2,6-diphenyl-piperidine, 5,10-diethoxy-2,3,7,8-tetrahydro-1H, and 6H-dipyrrolo[1,2-a; 1′,2′-d] pyrazine [150, 155]. They can also produce hydroxy fatty acids against a broad spectrum of food spoilage moulds [148, 149]. *L. hammesii* DSM 16381 was reported to produce monohydroxy C_18:1_ fatty acid with antifungal activities against *Mucor plumbeus*, *A. niger*, and *P. roqueforti* [156].

## 5. Conclusion and Recommendations

The harmful effects of mycotoxins in the food and feeds industry are numerous, ranging from health to economic losses. The mycotoxin menace has taken a toll on the African continent, as evidenced by the numerous episodes of aflatoxicosis aggravated by the warm weather and hence the need to come up with solutions that are native, economical, sustainable, and effective. LAB are naturally available in fermented foods in Africa and can be effective biological agents for controlling mycotoxins due to the wide variety of antifungal metabolites that they produce. The biological control of mycotoxins through LAB is a superior alternative over chemical and physical methods, which have negative health effects as LAB are generally regarded as safe, effective, and able to confer health benefits. LAB causes detoxification of mycotoxins through binding and absorption of the mycotoxins by the bacterial cell, where they are degraded and utilized as nutrient sources leading to the production of enzymes and other metabolites.

It is important that more strains of LAB are isolated from indigenous African foods and together with the known strains, they should be optimized for the detoxification of mycotoxins. More optimization also needs to be done to come up with the most effective consortia of LAB that are most effective against specific types of mycotoxins. An in-depth analysis of the LAB culture supernatants and separation of the compounds therein could give fractions of the compound that may be very effective against the mycotoxins in their pure state.

## Figures and Tables

**Table 1:** Mycotoxins and related fungi with commonly contaminating crops and foodstuffs in different African countries.

Mycotoxin	Foodstuffs	Related fungi	Country
Aflatoxins	Maize, milk, and animal feeds	*Aspergillus flavus* and *A. parasiticus*	Kenya
Aflatoxins	Groundnuts, cassava, millet, sorghum flour, and eshabwe sauce	*A. flavus* and *A. parasiticus*	Uganda
Aflatoxins	Meat products, spices, cereal grains, nuts and seeds, medicinal plants, milk, infant milk formula, cereals	*A. flavus* and *A. parasiticus*	Egypt
Nivalenol	Cereals and cereal products	*Fusarium* spp.	Tunisia and Morocco
Aflatoxins	Cereals and cereal products	*A. flavus and A. parasiticus*	
Ochratoxins A	Cereals and cereal products	*A. ochraceus, Penicillium verrucosum*	
Fumonisins	Cereals and cereal products	*Fusarium* spp.	
Fumonisins	Maize	*Fusarium* spp.	Tanzania
Aflatoxins	Maize	*A. flavus* and *A. parasiticus*	
Fumonisins	Maize	*A. flavus* and *A. parasiticus*	Zambia
Aflatoxins	Shiro, ground red pepper, sorghum, barley, teff and wheat sorghum, barley, and wheat	*A. flavus* and *A. parasiticus*	Ethiopia
Ochratoxins A	Sorghum	*A. ochraceus, P. verrucosum*	
Deoxynivalenol	Sorghum	*Fusarium* spp.	
Fumonisins		*Fusarium* spp.	
Zearalenone		*Gibberella* spp. and *Fusarium* spp.	
Aflatoxins	Sesame oil, groundnut oil, and peanuts butter	*A. flavus* and *A. parasiticus*	Sudan
Aflatoxins	Rice and weaning foods	*A.flavus* and *A. parasiticus*	Nigeria
Ochratoxins A	Rice	*A. ochraceus, P. verrucosum*	
Aflatoxins	Maize	*A. flavus* and *A. parasiticus Fusarium* spp.	Ghana
Fumonisins	Maize		
Aflatoxins	Maize	*A. flavus* and *A. parasiticus*	Benin
	Chips		
Aflatoxins	Dried vegetables (baobab leaves, hot chili, and okra)	*A. flavus* and *A. parasiticus*	Benin, Mali, and Togo
Aflatoxins	Groundnuts	*A. flavus* and *A. parasiticus*	Burkina Faso
Fumonisins	Maize and compound feeds	*Fusarium* spp.	South Africa
Deoxynivalenol	Compound feeds	*Fusarium* spp.	
Zearalenone	Compound feeds	*Gibberella* spp. and *Fusarium* spp.	
Zearalenone	Sorghum beer	*Gibberella* spp. and *Fusarium* spp.	Lesotho

Adapted from [29] with modifications.

**Table 2:** Common lactic acid-fermented cereal-based food products in Africa.

Product	Substrate	LAB	Nature of use	Region	Reference
Seketeh	Maize	*Lactobacillus plantarum, Lactococcus lactis*	Alcohol	Nigeria	[98]
Sorghum beer	Sorghum, maize	LAB	Liquid drink, acidic, weakly alcoholic drink	South Africa	[98]
Tobwa	Maize	LAB	Nonalcoholic drink	Zimbabwe	[98]
Uji	Sorghum, maize, millet	*L. paracasei*	Nonalcoholic	Kenya, Uganda, Tanzania	[99]
Pito	Maize, sorghum, and millet	*Leuconostoc mesenteroides*	Alcoholic dark brown drink	Nigeria, Ghana	[100]
Ogi	Maize, millet sorghum	*L. plantarum*	Paste as staple, weaning food for babies alcoholic	Nigeria, West Africa	[100]
Nasha	Sorghum	LAB	Porridge	Sudan	[98]
Mutwiwa	Maize	LAB	Porridge	Zimbabwe	[98]
Merissa	Sorghum and millet	LAB	Alcoholic	Sudan	[98]
Mahewu	Maize	*Lc. lactis* subsp*. lactis*	Solid staple	Zimbabwe	[101]
Mawe	Maize	LAB	Basis for preparation of many dishes	South Africa	[98]
Kunun-zaki	Maize, sorghum, and millet	*L. fermentum, L. leichimani, Streptococcus* spp.	Paste used as a breakfast dish	Nigeria	[100]
Kisra	Sorghum	LAB	Staple as bread	Sudan	[100]
Koko	Maize	*L. plantarum, Lb. brevis*	Porridge as staple	Ghana	[98]
Kenkey	Maize	*L. fermentum, L. reuteri*	Mush	Ghana	[98]
Kaffir beer	Kaffir corn, rice	LAB	Alcoholic drink liquid	South Africa	[98]
Injera	Sorghum, tef, maize or wheat	LAB	Bread-like staple	Ethiopia	[102]
Ilambazi lokubilisa	Maize	LAB	Porridge as weaning food	Zimbabwe	[98]
Doro	Finger millet malt	LAB	Colloidal thick alcoholic drink	Zimbabwe	[98]
Banku	Maize	LAB	Dough as staple	Ghana	[98]
Burukutu	Sorghum	*Leuc. Mesenteroides*	Alcoholic drink	Nigeria, Benin, Ghana	[103]
Busaa	Rice, millet, maize	*L. helveticus, Lb. salivarus, Lb. casei, Lb. brevis, L. plantarum, Lb. buchneri,*	Alcoholic	Nigeria, Ghana	[98]
Fura	Maize and sorghum	*L. plantarum, Pediococcus, Leuconostoc, Streptococcus, Enterococcus*		Nigeria	[100]
Agidi	Maize, sorghum and millet	LAB		Nigeria	[98, 100]
Burukutu	Sorghum and millet	*Leuc. mesenteroides*		West Africa	[100]
Bushera	Sorghum and millet	LAB	Nonalcoholic	Uganda	[104]
Tella	Barley, wheat, sorghum, millet, maize, and teff	LAB	Alcoholic	Ethiopia	[105]

**Table 3:** Biodetoxification of mycotoxin by LAB strains.

Mycotoxins	LAB	Mechanism of detoxification	Reference
AFB1	*L. plantarum* MON03	Binding	[115]
	*L. plantarum* C88	Binding	[116]
	*L. kefiri* KFLM3	Adsorption and biotransformation	[117]
	*L. acidophilus* and *L. brevis*	Binding	[118]
	*L. rhamnosus* yoba 2012	Binding	[119]
	*L. fermentum* TMU121 and *Pediococcus pentosaceus* TMU457	Binding	[120]
	*L. casei, L. plantarum,* and *L. fermentum*	Binding	[121]
	*L. reuteri* ŁOCK 1096 and *L. casei* ŁOCK 0911	Binding	[122]
	*P. pentosaceus* L6, *L. plantarum* L12, *Leuconostoc mesenteroides* L18, *L. coryniformis* subsp. *coryniformis* L47, *L. brevis* L52	Binding and adsorption	[123]
	*Enterococcus faecium M74*	Binding	[124]
AFM1	*L. plantarum* MON03 and *L. rhamnosus* GAF01	Binding	[125]
	*L. delbrueckii* spp. *bulgaricus* LB340, *L. rhamnosus* HOWARU, and *Bifidobacterium lactis* FLORA-FIT BI07	Binding	[126]
	*L. bulgaricus* and *Streptococcus thermophiles*	Binding	[127]
	*L. plantarum* ATCC 10697, *B. animalis* ATCC 27672, and *B. bifidum* ATCC 35914	Binding	[128]
OTA	*L. acidophilus* VM 20	Binding	[129]
	*L. rhamnosus CECT 278T* and *L. plantarum CECT 749*	Adsorption	[85]
PAT	*Bifidobacterium animalis* VM 12	Binding	[129]
	*Enterococcus faecium M74*	Binding	[124]
DON	Various LAB	Adsorption	[130]
	*L. plantarum* LP102	Binding	[131]
	*L. paracasei LHZ-1*	Adsorption	[132]
ZEN	*L. pentosus*	Adsorption	[133]
	*L. plantarum*	Binding	[134]
	*L. rhamnosus GG/LC705*	Binding	[64]
FB1/B2	*L. brevis, L. plantarum, L. paracasei, L. casei, L. pentosus, L. reuteri*, and *L. rhamnosus*	Binding	[122]
	*L. plantarum* B7 and *L. pentosus X8*	Binding	[135]
T-2 toxin	*L. plantarum* LP102	Binding	[131]

AFB_1_: aflatoxins B_1_; AFM_1_: aflatoxin M_1_; OTA: ochratoxin A; PAT: patulin; DON: deoxynivalenol; ZEN: zearalenone; FB_1_/B_2_: fumonisin B_1_/B_2_; T-2 toxin: trichothecenes-2 toxin [79].

**Table 4:** Lactic acid bacteria and their active compounds against mycotoxin-producing fungi.

LAB strain	Activity spectrum	Active compounds	Reference
*Lactobacillus casei*, *L. rhamnosus*, *L. fermentum, L. acidophilus*, *L. plantarum, L. sakei*, and *L. reuteri*	*Penicillium expansum* and *Aspergillus flavus*	3-Phenyllactic acid	[143]
*L. sanfranciscensis* CB1	*Fusarium* spp., *Penicillium* spp., *Aspergillus* spp., *Monilia* spp.	Caproic acids, propionic acid, butyric acid, n-valeric acid	[144]
*L. plantarum* 21B	Broad-spectrum	4-Hydroxy-phenyllactic acids	[145]
*L. plantarum* MiLAB 393	Broad-spectrum	3-Phenyllactic acid, cyclo(Phe-Pro), cyclo(Phe-OH-Pro)	[146]
*L. coryniformis* Si3	Broad-spectrum	Cyclic dipeptides, phenyllactic acid, cyclo(Phe-Pro), cyclo(Phe-OH-Pro)	[147]
*L. plantarum* MiLAB 14	Broad-spectrum	Hydroxy fatty acids	[148]
*L. plantarum* K35	*A. flavus* and *A. parasiticus*	Lactic acid, 2-butyl-4-hexyloctahydro-1H-indene, oleic acid, palmitic acid, linoleic acid, 2,4-di-tert-butylphenol, stearic acid, 3-phenyllactic acid, and pyroglutamic acid	[149]
*L. plantarum* FST 1.7	*F. culmorum* and *F. graminearum*	Lactic acid, phenyllactic acid, cyclic dipeptides cyclo(l-Leu-l-Pro), and cyclo (l-Phe-l-Pro)	[150]
*L. plantarum* AF1	*A. flavus*	C_12_H_22_N_2_O_2_, 3,6-bis(2-methylpropyl)-2,5-piperazinedion	[151]
*L. pentosus* TV35b	*Candida albicans*	Pentocin TV35b	[152]
*L. plantarum* VE56, *Weissella cibaria* FMF4B16, and *W. paramesenteroides* LC11.	Broad-spectrum	Phenyllactic acid and 2-hydroxy-4-methylpentanoic acid	[153]
*L. plantarum* IMAU10014	Broad-spectrum	3-Phenyllactic acid, benzeneacetic acid, and 2-propenyl ester	[154]
*L. casei* AST18	*Penicillium* sp.	Cyclo-(Leu-Pro), 2,6-diphenyl-piperidine, and 5,10-diethoxy-2,3,7,8-tetrahydro-1H, 6H-dipyrrolo[1,2-a; 1′,2′-d] pyrazine	[155]
*L. hammesii* DSM 16381	*Mucor plumbeus*, *A. niger*, and *P. roqueforti*	Monohydroxy C_18:1_ fatty acid	[156]
*L. reuteri* ee1p	*P. expansum*, *Trichophyton tonsurans*	(S)-(-)-2-hydroxyisocaproic acid, hydrocinnamic acid, phenyllactic acid, decanoic acid, azelaic acid, 4-hydroxybenzoic acid, p-coumaric acid, vanillic acid, DL-b-hydroxyphenyllactic acid, and 3-hydroxydecanoic acid	[157]
*L. amylovorus* DSM 19280	*A. fumigatus* and *F. culmorum*	Carboxylic acids, cinnamic acid derivatives (3-phenylpropanoic acid, p-coumaric, and (E)-2-methylcinnamic acid), 3-phenyllactic acid and its hydroxy derivative (3-(4-hydroxyphenyl), lactic acid, acetic acid, D-glucuronic acid and salicylic acid, nucleosides (cytidine and 2′-deoxycytidine), sodium decanoate, and cyclic dipeptides	[158]
*L. plantarum* LB1 and *L. rossiae* LB5	*P. roqueforti* DPPMAF1	Organic acids (formic acid) and four antifungal peptides	[159]
*L. plantarum* TE10	*A. flavus* MD3	Two antifungal peptides (VLHEPLF and ALKAAPSPA)	[160]
*Lactobacillus* sp. RM1	*A. parasiticus*	6-Octadecenoic acid methyl ester, hexadecanoic acid methyl ester, phenol, 2,4-bis(1,1-dimethylethyl), (Z)-7-hexadecenal, pentadecane, dotriacontane, and 2-methyldecane	[142]
*L. plantarum* UM55	*A. flavus*, *A. parasiticus*, *A. arachidicola*, *A. nomius,* and *A. minisclerotigenes*	Lactic acid, phenyllactic acid, hydroxyphenyllactic acid, and indole lactic acid	[161]
*L. reuteri*	*A. niger*	n-Decanoic acid, 3- hydroxydecanoic acid and 3-hydroxydodecanoic acid	[162]
*L. fermentum* CRL 251	*A. niger, Penicillium* sp., and *F. graminearum*	Lactic, acetic, and phenyllactic acids	[163]
*Leuconostoc citreum* L123, *L. brevis* Lu35, *L. reuteri* 5529, *L. spicheri* O15, and *Propionibacterium freudenreichii* LSaci68	*P. corylophilum* and *A. niger*	Lactic, acetic, and propionic acids, ethanol and hydrogen peroxide, phenyllactic, hydroxyphenyllactic, azelaic, and caproic acids	[164]
*L. plantarum* UFG 121	Broad-spectrum	Lactic acid and phenyllactic acid	[165]

## Data Availability

The reference data used to support the findings of this study are included within the article.
